# Growth Performance, Carcass Quality and Gut Microbiome of Finishing Stage Pigs Fed Formulated Protein-Energy Nutrients Balanced Diet with Banana Agro-Waste Silage

**DOI:** 10.3390/life15071033

**Published:** 2025-06-28

**Authors:** Lan-Szu Chou, Chih-Yu Lo, Chien-Jui Huang, Hsien-Juang Huang, Shen-Chang Chang, Brian Bor-Chun Weng, Chia-Wen Hsieh

**Affiliations:** 1 Department of Agricultural Biotechnology, National Chiayi University, Chiayi 600, Taiwan; choulb@mail.ncyu.edu.tw; 2 Department of Food Sciences, National Chiayi University, Chiayi 600, Taiwan; chihyulo@mail.ncyu.edu.tw; 3 Department of Plant Medicine, National Chiayi University, Chiayi 600, Taiwan; chienjui.huang@mail.ncyu.edu.tw; 4 Southern Region, Taiwan Livestock Research Institute, Ministry of Agriculture, Pingtung 912, Taiwan; hjhuang@tlri.gov.tw (H.-J.H.); macawh@tlri.gov.tw (S.-C.C.); 5 Department of Microbiology, Immunology and Biopharmaceuticals, National Chiayi University, Chiayi 600, Taiwan; brian@mail.ncyu.edu.tw

**Keywords:** banana agro-waste, silage, pigs, growth performance, carcass quality, gut microbiota, COG annotation, sustainable feed

## Abstract

This study evaluated the effects of fermented banana agro-waste silage (BAWS) in finishing diets for KHAPS pigs (Duroc × MeiShan hybrid). BAWS was produced via 30 days of anaerobic fermentation of disqualified banana fruit, pseudostem, and wheat bran, doubling crude protein content and generating short-chain fatty acids, as indicated by a satisfactory Flieg’s score. Thirty-six pigs were assigned to control (0%), 5%, or 10% BAWS diets formulated to meet NRC nutritional guidelines. Over a 70-day period, BAWS inclusion caused no detrimental effects on growth performance, carcass traits, or meat quality; a transient decline in early-stage weight gain and feed efficiency occurred in the 10% group, while BAWS-fed pigs demonstrated reduced backfat thickness and increased lean area. Fore gut microbiome analysis revealed reduced *Lactobacillus* and elevated *Clostridium sensu stricto 1*, *Terrisporobacter*, *Streptococcus*, and *Prevotella*, suggesting enhanced fiber and carbohydrate fermentation capacity. Predictive COG (clusters of orthologous groups)-based functional profiling showed increased abundance of proteins associated with carbohydrate transport (COG2814, COG0561, COG0765) and stress-response regulation (COG2207). These results support BAWS as a sustainable feed ingredient that maintains production performance and promotes fore gut microbial adaptation, with implications for microbiota-informed nutrition and stress resilience in swine.

## 1. Introduction

Banana is the leading export fruit, with approximately 10,664 hectares under cultivation, producing around 300,000 metric tons annually and generating an economic value of roughly NT$120 million [1]. Bananas have been identified as effective carbon-capturing and storage crops, sequestering between 0.37 and 4.1 t/ha per cultivation cycle. However, banana cultivation also generates a carbon footprint of about 1.2 kg CO_2_ per kg of bananas produced, primarily due to agro-waste disposal [2]. Thus, the benefits of carbon recycling in banana agriculture are realized only when agro-waste—mainly pseudo-stem, leaves, and unmarketable fruit—is properly managed or valorized. Among proposed solutions, converting banana waste into biofuel has garnered significant attention, although economic viability remains a major challenge. Another promising avenue is the use of banana agro-waste as animal feed silage, which has been extensively investigated in ruminants [3,4]. The study in non-ruminant animals, such as swine fed with the high-fibrous banana agro-waste, remains to be better elucidated.

Recently, solid state fermentation of banana peel waste with fungal species for 7 days has been shown to reduce the contents of total carbohydrate, phenolics, flavonoids, and vitamin C but increase protein level [5]. Earlier, solid state fermentation using yeast *Saccharomyces cerevisiae* or *Rhizopus oryzae* resulted in a similar 4% increase in protein contents after 5- and 1-day fermentation, respectively [6]. This suggests that microbial fermentation of fibrous banana agro-waste can enhance protein content and antioxidant phenolics through biotransformation.

Recent studies [7,8] using finishing stage LYD pigs fed a reduced soybean meal 12% low-protein diet (normally 16% crude protein diet), their results demonstrated adverse effects of low-protein diets could be reversed by supplementing with 0.57% glycine. Metabolomic analysis on muscle samples revealed that glycolysis, AMP/ADP/UMP levels, and oxidative enzyme activity were significantly lower in the low-protein group compared to the normal group. Changes in carbohydrate energy utilization and protease activity may affect meat quality. Supplementing glycine in low-protein diets might support biotransformation of gut microbiome in improved carcass quality and overall growth performance. In nursing pigs, changes in protein sources from casein-based milk to plant-source dry feed often lead to gastrointestinal complications. Common pathogens include *Escherichia coli*, *Bacteroides*, and *Clostridium* species [9]. Moreover, according to Gao et al. [10], when weaning piglets were provided with 17% (normal) or 30% casein-based diets, high-protein diets caused diarrhea, colonic content analysis showed a substantial reduction in short-chain fatty acids, with acetate and tri-methyl-butyrate particularly reduced. These differences suggest that changes in gut microbial composition and diversity impact the production of metabolites, which in turn have significant implications for host energy and protein utilization.

After decades of breeding selection and foundational support for growth traits, the three-line hybrid LYD pig breed has become a globally prevalent pork production breed. The pig farming industry is intricately tied to the nutritional utilization of this breed, as feed constitutes more than 50% of the total production costs [8]. Generally, soybeans and corn are the main sources of digestible protein and energy in feed composition. However, this traditional nutritional formula, which focuses on gastrointestinal digestion and absorption, may significantly underestimate the role of gut microbiota in complex protein content or fibrous material utilization. With the maturity and accessibility of omics research methods, constructing large-scale omics databases to associate breed genomic traits with growth and health-related metabolomes and microbiomes has become feasible.

In this study, we focus on the KHAPS breed pigs—derived from seven generations of paternal Duroc crossed with a maternal native *Sus scrofa domestica* (MeiShan) breed [11]—to evaluate banana agro-waste silage (BAWS) as a dietary supplement. We aim to determine its effects on growth performance, carcass and meat quality, and associated gut microbiome dynamics.

## 2. Materials and Methods

### 2.1. Banana Agro-Waste Silage Preparation

Banana (*Musa Cavendish* AAA) agro-waste, including disqualified banana, pseudostem, leaves, and rachis. The disqualified banana and pseudostem were recycled and utilized as raw materials in the current study. Initial preparation was chopping and mixing disqualified banana, pseudostem, and wheat bran at a ratio of 3:2:1. Subsequently, the mixture was subjected to an anaerobic fermentation (room temperature 25–30 °C) for 30 days to produce banana agro-waste silage (BAWS). The nutritional value of the BAWS was analyzed by the Feed Analysis Center of the Livestock Research Institute, Council of Agriculture, Taiwan (https://english.tlri.gov.tw/view.php?theme=web_structure&id=179). The final silage product of three different batches was subjected to analysis of pH, dry matter, and short-chain fatty acids, including acetic acid, butyric acid, and lactic acid. The silage quality assessment with Flieg’s score was performed according to the methodology of Flieg, 1938 [12].

### 2.2. Animal Experimentation

All the procedures used in this experiment were approved by the Institutional Animal Care and Use Committee of Taiwan Livestock Research Institute, Ministry of Agriculture (TLRI IACUC, protocol No. 113021, 2023). Thirty-six KHAPS (Kaohsiung Animal Propagation Station) black pigs, which were derived from seven generations cross cross-breeding of paternal Duroc and a maternal native *Sus scrofa domestica* breed (MeiShan) [11], including an equal number of both gilts and barrows at their finishing period, were randomly assigned into three treatments. Each treatment had three replicates, as different pens were allocated in a feeding house. The average temperature was 26.4 °C (25.4 to 28.5 °C) and the humidity was 81.2% (80.3 to 82.2%) during the period of experimentation. Feed and water were provided throughout the entire experiment. The treatments were a control group of a formulated balanced diet (0%), and 5% or 10% corn substitution with BAWS groups (Table 1). Proximate feed analysis and amino acid analyses were conducted using the methodology of Huang et al. [13]. All diets were formulated to satisfy finishing stage pig nutrition requirements according to NRC, 1998 [14].

### 2.3. Feed Intake and Growth Performance

Body weight (BW) and feed intake (FI) were recorded every week to monitor weight gain and feed conversion throughout the experiment. Pigs’ body weight (BW) and feed intake (FI) were calculated to obtain average daily gain (ADG), average daily feed intake (ADFI) on a dry matter basis, and feed conversion ratio (FCR). The FCR values were calculated using the following formula:

Feed conversion ratio (FCR) = (ADFI/ADG) × 100

### 2.4. Carcass and Meat Quality

Pigs were fasted for 24 h with access to clean water prior to slaughter at a commercial slaughterhouse. All pigs were stunned using electrical stunning in accordance with humane slaughter procedures before exsanguination. Carcasses were processed under standard hygienic conditions, and samples were collected following chilling. Each of the 3 pigs from the same treatments, including two barrows and one gilt, was selected and processed according to standard operating procedures for carcass and meat quality assessments. Briefly, carcass weight was measured, and the carcass was chilled at 0–4 °C for 24 h. Carcass was then segmented according to the cutting specifications outlined by the Taiwan Meat Development Foundation (1992). Post-slaughter pH values, sensory evaluation, and longissimus dorsi chemical composition were performed. Muscle samples were collected for chemical composition analysis, and moisture, ash, crude fat, and crude protein contents were determined following AOAC methods (2000) [15].

### 2.5. Metagenomics Analysis of the Gut Microbiome

#### 2.5.1. Sample Collection and DNA Extraction

During the slaughter process, the digestive tract was promptly separated. For the fore gut sample, a 20 cm segment of the upper intestine (duodenum) was dissected and collected. The intestinal segment was longitudinally opened using sterilized scissors, and the luminal contents were obtained by scraping the internal wall with a sterile spatula into a 50 mL centrifuge tube. The samples of the hind gut were digesta from a segment of the colon immediately connected to the rectum. The collected fore and hind gut contents were immediately preserved in a dry-ice container and subsequently transferred to a −80 °C laboratory freezer for storage until further analysis.

For microbiota profiling, gut samples were collected from three animals per group, including one female and two castrated males. After thawing, each gut content sample was thoroughly homogenized, and a 0.5 mL aliquot was used for genomic DNA extraction. Microbial DNA was extracted using the MO BIO PowerMag^®^ Soil DNA Isolation Kit (Carlsbad, CA, USA), following the manufacturer’s protocol. The extracted DNA samples were stored at −80 °C until further molecular analyses.

#### 2.5.2. Metagenome Library Preparation and Sequencing

The 16S rRNA gene was amplified using region-specific primers appended with Illumina overhang adapter sequences. Each PCR reaction was carried out in a 25 μL reaction volume containing 12.5 μL of KAPA^®^ HiFi HotStart ReadyMix (KAPA BIOSYSTEMS, Wilmington, MA, USA), 5 μM of both forward and reverse primers, and approximately 20 ng of template DNA. The PCR conditions were as follows: initial denaturation at 95 °C for 3 min; 25 cycles of denaturation at 95 °C for 30 s, annealing at 55 °C for 30 s, and extension at 72 °C for 30 s; with a final extension step at 72 °C for 5 min. First-round amplicons were purified using the AMPure XP system (Beckman Coulter, Indianapolis, IN, USA), followed by indexing with the Nextera XT Index Kit v2 Set (Illumina, San Diego, CA, USA). The quality and concentration of the indexed libraries were evaluated using a Qubit™ 2.0 Fluorometer (Thermo Scientific, Waltham, MA, USA) and a Qsep400 system equipped with the N1 High Sensitivity Cartridge Kit (Bioptic Inc., Xindian Dist., NTC, Taiwan).

The qualified libraries were sequenced on an Illumina MiSeq platform using 300 bp paired-end reads. Sequencing was performed by Genomics, BioSci & Tech Co. (New Taipei City, Taiwan).

#### 2.5.3. Bioinformatics Analysis

Amplicon sequencing of the 16S rRNA V3–V4 region and the ITS region was performed on the Illumina MiSeq platform (2 × 300 bp paired-end reads) by Genomics BioSci & Tech Co. (New Taipei City, Taiwan). Raw reads were initially quality-trimmed using Trimmomatic (v0.39) [16] and demultiplexed with an in-house script. Primer sequences (341F–805R for 16S or ITS-specific primers) were trimmed using Cutadapt (v3.5) [17], with reads shorter than 150 bp or exceeding an error rate of 0.1 removed.

Amplicon sequence variants (ASVs) were generated using the DADA2 pipeline (v1.22) [18], including quality filtering, denoising, paired-end merging, and chimera removal. Taxonomic classification was performed in QIIME2 (v2021.8) [19] using a Naive Bayes classifier trained on the SILVA 138 database (99% identity) [20] for 16S data, and on the UNITE database for ITS sequences.

Alpha diversity indices, including Chao1, Observed Species, Good’s Coverage, and Fisher’s Alpha (species richness), as well as Shannon, Simpson, and ENSPIE (species evenness), were calculated. Beta diversity was assessed using unweighted and weighted UniFrac distances, and visualized through hierarchical clustering, heatmaps, principal coordinate analysis (PCoA), and principal component analysis (PCA) based on Bray–Curtis dissimilarity.

Taxonomic composition was visualized via stacked bar plots and interactive KRONA charts (v2.7.1) [21], while Venn diagrams (VennDiagram, v1.6.20) illustrated shared and unique ASVs across sample groups.

To infer microbial functional potentials, two complementary tools were used: PICRUSt2 and Tax4Fun2. PICRUSt2 was applied to predict Kyoto Encyclopedia of Genes and Genomes (KEGG) orthologs and metabolic pathway abundances based on 16S rRNA sequences [22]. In parallel, Tax4Fun2 was employed to provide an alternative functional annotation based on SILVA-derived taxonomic profiles [23]. Predicted gene functions and pathway-level differences among groups were further analyzed and compared.

### 2.6. Statistics

Data on growth performance were analyzed using the GLM (General Linear Model) procedure. Treatment effects were compared via one-way ANOVA, followed by a Tukey post hoc test. Statistical significance was set at *p* value < 0.05. Treatment data were presented as mean with a sample size of *n* = 12 per treatment group. Carcass, meat quality, and microbiome analysis were conducted on the data collected from 3 pigs, including 1 gilt and 2 barrows, from each group. The relative abundance of both fore and hind gut microbiomes at phylogenetic levels of family and genus was calculated and shown. Data were the mean of three pigs from each treatment (gender effect was not compared), and treatment comparison was using SigmaPlot 12.5.

## 3. Results

Gut microbiome dynamics have evolved to reflect the food sources and rapidly adapt to food accessibility in order to maximize the utilization of available nutrients for host co-evolved accountability. Founded on the success of life-dependent law, feeding of BAWS substituted diet at the level of 0, 5, and 10% in fishing-stage pigs on a formulated nutrients-balanced diet has revealed a prevalence shift in unique gut microbes. The association in alterations of body composition via carcass assessments has been addressed, and to provide an outlook in promoting a sustainable one health of people, animals, and the ecosystem.

### 3.1. Feeding Value of BAWS

Banana (*Musa Cavendish AAA*) agro-waste silage (BAWS) obtained from a 30-day anaerobic fermentation of disqualified banana, pseudostem, and wheat bran (in a 3:2:1 ratio) was assessed for nutritional value as listed in Table 2.

BAWS exhibited markedly improved nutritional profiles. Crude protein (CP) increased to 12.43%, nearly double that of disqualified banana (6.68%) and pseudo-stem (6.10%). Amonium nitrogen level was increased to 1911.6 ppm, 12 times increased than compared with those of disqualified banana (139.7 ppm) or pseudostem (158.1ppm). BAWS contained 28.08% neutral detergent fiber (NDF), 15.24% acid detergent fiber (ADF), and 2.33% acid detergent lignin (ADL), which indicates that fermentation partially degraded the cell-wall components. Water-soluble carbohydrate (WSC) content declined to 1.40% in BAWS, compared with 3.83% in disqualified banana and 17.23% in pseudo-stem, reflecting extensive microbial consumption of readily fermentable carbohydrates. Total organic carbon (TOC) remained stable (BAWS: 54.79%; raw materials: 51.85–55.09%), indicating consistent retention of organic matter. Fermentation quality was further confirmed by a final pH of 4.33 ± 0.27, along with acetic acid at 4.41 ± 0.26% and lactic acid at 2.46 ± 0.69%, while butyric acid was not detected. The Flieg’s silage quality score of 54.0 ± 3.0 classified BAWS as “satisfactory” (>40). Moreover, BAWS exhibited a light-brown to yellowish appearance, and its mild acidic aroma was considered acceptable.

In summary, compared to its raw ingredients, BAWS demonstrates significantly enhanced protein and nitrogen contents, partial fiber breakdown, and favorable fermentation characteristics, supporting its viability as an improved feed supplement.

### 3.2. Dietary Intervention with BAWS Formulated Feed in Finishing Stage Pigs

A complete randomized design to study the effect of different levels of BAWS formulated into nutrient nutrient-balanced complete diet in finishing stage pigs was studied.

#### 3.2.1. Growth Performances

Body weights during the entire 70-day finishing period, measured at day 0, 21, 42, and 70, did not differ significantly among the control, 5% BAWS, and 10% BAWS groups (*p* > 0.05) (Table 3). However, the 10% BAWS group showed significantly (*p* < 0.05) lower weight gain at the periods of 21–42 and 42–70 as compared with the control group. In addition, the daily feed intake during the initial period of 0–21 days, and the feed conversion ratio at periods of 21–42 and 42–70 days were significantly (*p* < 0.05) different in the 10% BAWS group than the 5% BAWS and control groups. Nevertheless, no significance (*p* > 0.05) was obtained among the three groups in comparisons of daily weight gain, daily feed intake, and feed conversion ratio of the entire 0–70 days finishing stage.

#### 3.2.2. Improvement of Carcass Characteristics and Lean Composition, and Sensory of Taste

As shown in Table 4, the average live body weight and carcass weight were not significantly different (*p* > 0.05) among groups. Moreover, back fat thickness and thickness measured at three skinless points of the first rib, last ribs, and lumbar were on average lower in pigs of the BAWS groups than the control group.

Similarly, meat quality was not significantly (*p* > 0.05) different in meat color score, marbling score, firmness score, and lumbar meat area. Nevertheless, the lumbar meat area of the longissimus muscle section showed about a 20% increase in the BAWS groups as compared with that of the control group. In the sensory score, there were significant differences in flavor, juiciness, and tenderness among groups.

### 3.3. Metagenomics Analysis of Gut Microbiomes via Next Generation Sequencer

**Figure 1:**
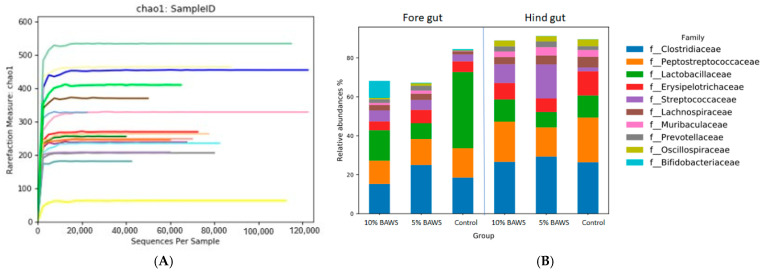
Gut microbiome analysis validation with the Rarefaction curve measure (**A**), and the top 10 taxa at the family level of fore gut and hind gut in three experimental groups (**B**).

The gut microbiome analysis, conducted using 16S next-generation sequencing, demonstrated high-quality reads with Phred scores above 30. Rarefaction curves for all samples reached a plateau, indicating sufficient sequencing depth (Figure 1A). Figure 1B illustrates the ten most abundant bacterial families in both the fore gut and hindgut across the control, 5% BAWS, and 10% BAWS groups. Taxonomic analysis r revealed that seven of the top ten most abundant bacterial families were in the phylum Bacillota. These families were *Clostridiaceae*, *Peptostreptococcaceae*, *Lactobacillaceae*, *Erysipelotrichaceae*, *Streptococcaceae*, *Lachnospiraceae*, and *Oscillospiraceae*, and together they accounted for more than sixty percent of the total relative abundance. The remaining three families were *Prevotellaceae* and *Muribaculaceae*, which belong to the phylum Bacteroidota, and *Bifidobacteriaceae*, which belongs to the phylum Actinomycetota. In the fore gut of the control group, *Lactobacillaceae* was the highest relative abundance family, and only a trace of *Prevotellaceae* when compared with those of the BAWS groups. Additionally, the enriched *Bifidobacteriaceae* were noticed in the foregut of the 10% BAWS group but not in the others. Both *Streptococcaceae* and *Prevotellaceae* showed consistently higher relative abundances in both fore gut and hind gut of BAWS groups compared to controls.

#### 3.3.1. Fore Gut—Reduction in the Abundance of Genus Lactobacillus

**Figure 2:**
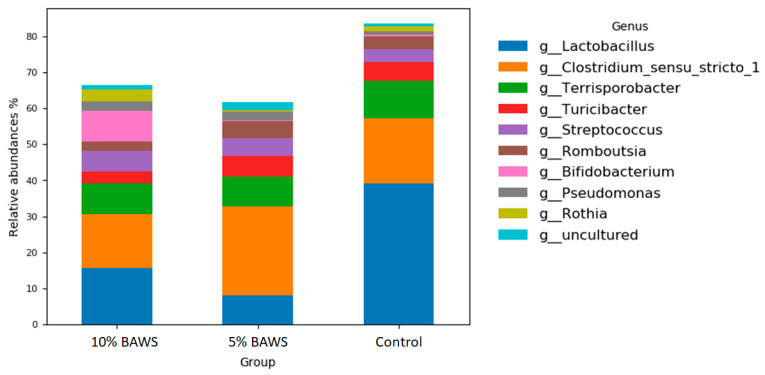
The changes in relative abundance of fore gut microbiome at the genus level in pigs fed different levels of dietary BAWS.

Figure 2 shows the fore gut microbiome at the genus level of the three groups. The relative abundance of the genus *Lactobacillus* was largely reduced in both 10% and 5% BAWS groups as compared with the control group. Along with the reduction, the populations had shifted primarily to the genus of *Clostridium* in 5% BAWS group, and to the genus of *Bifidobacterium* (Pink), *Streptococcus* (Purple), *Pseudomonas* (Gray), and *Rothia* (Olive) in the 10% BAWS group. In addition, the dietary 10% BAWS group exhibited decreases in *Terrisporobacter* (Green), *Turicibcter* (Red), and *Romboutsia* (Brown).

#### 3.3.2. Hind Gut—Increase in the Abundance of *Streptococcaceae* and *Prevotellaceae* Families

**Figure 3:**
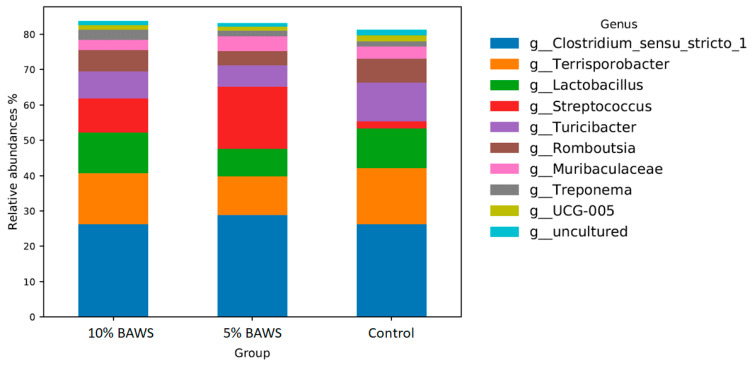
The changes in relative abundance of hind gut microbiome at the genus level in pigs fed different levels of dietary BAWS.

In the hind gut, *Clostridium* was the dominant genus across all treatment groups, as shown in Figure 3. In the 5% BAWS group, *Streptococcus* was the second most abundant genus, while *Terrisporobacter* occupied this position in both the 10% BAWS and control groups. Dietary BAWS groups had reduced abundances of *Turicibacter* and *Romboutsia* as compared to the control group. In general, the microbiome of the hind gut remains relatively consistent across groups in a nutrient-balanced diet.

#### 3.3.3. Predicting the Functional Distribution of Homologous Proteins with Similar Functions and Interpreting Their Significance

**Figure 4:**
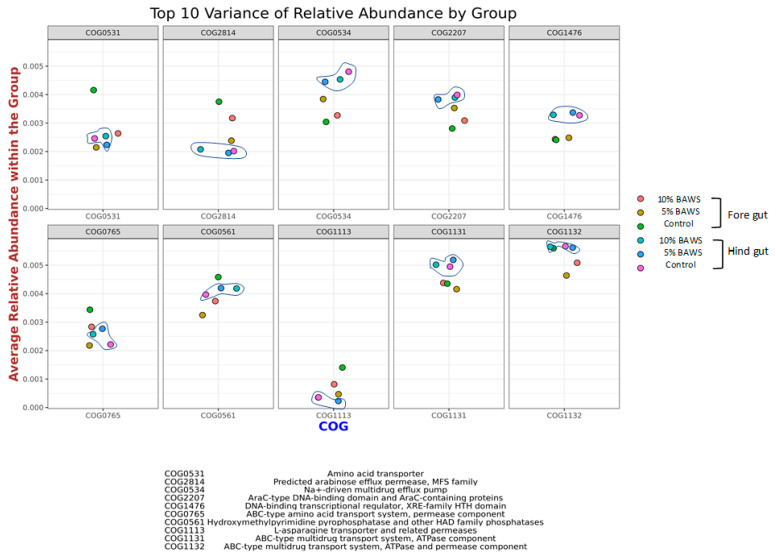
Average relative abundance of phylogenetic lineages of COGs in the fore gut and hind gut (grouped with line drawing) within the treatment groups.

The Clusters of Orthologous Groups (COG) database classifies the proteins based on sequence and functional similarity of top 10 variance of relative abundance at genome-scale analyses in fore gut and hind gut is shown (Figure 4). The relative abundance of the top 10 variance in the COG cluster proteins among groups included COG0531, COG2814, COG0534, COG2207, COG1476, COG0765, COG0561, COG1113, COG1131, and COG1132. In general, the patterns of the top 10 variance of relative abundance protein by grouping exhibit less difference in hind gut groups (circle in line drawing) as compared to fore gut groups. The average abundance of COG0534, COG2207, COG1476, COG1131, and COG1132 was higher in hind gut groups than those of fore gut. Conversely, the COG2814 and COG1113 had an average higher abundance within the group in fore gut than the hindgut. Furthermore, in the fore gut result, the 5% BAWS group had higher abundance (close to the clusters of hind gut) in COG0534 and COG2207, but lower abundance in COGCOG2814, COG0765, and COG1113.

## 4. Discussion

### 4.1. Feed Banana Agro-Waste Silage Had No Impact on Growth, Carcass, and Meat Quality

Taiwan’s sustainable agriculture crucially relies on resource recycling and cross-utilization among the vital small-holder farms. Banana agriculture produces magnificent waste, including mainly the pseudostem and disqualified banana. Nutritional content of disqualified banana and pseudosteam (Table 2) of *Musa Cavendish AAA* revealed more than 50% total organic carbon (TOC) and about 6% crude protein. Previous studies [24,25] have reported that the disqualified banana (primarily immature green banana) had around 60 to 80% starch content (dry basis), including more than 30% resistant starch, which exhibits metabolic regulation through gut flora, and other healthy ingredients such as vitamins and phytochemcials. The health benefits of green banana consumption have been systematically reviewed in humans [26] and showed regulatory functions on gastrointestinal symptoms, glycocemic homeostasis, weight control, hepatoprotection, renal integrity, and general metabolic complications. In domestic animals, previously, fresh banana agro-waste fed to goat and cattle at 10% inclusion into TMR (total mixed ration) had promised results in growth and slaughter efficiency (not published). Yet, the nutritional value is relatively low when considering its protein level. To expand the feeding-grade recycling to pigs, banana agro-waste was subjected to a simple anaerobic microbial biotransformation, and it was demonstrated that the crude protein (CP) of the BAWS had doubled. Moreover, the ammonium content had more than a 10 times increase, which suggested the bioavailable nitrogen derived from microbial fermentation.

In the current pioneer feeding study, anaerobic fermentation increased the crude protein (CP) content of banana agro-waste from approximately 6% to 12%, yielding the final product BAWS. BAWS was included at up to 10%, replacing corn and with added wheat bran, to formulate a finishing diet containing 15.5% CP, 0.85% lysine, 3250 kcal/kg metabolizable energy, and approximately 1.9% crude fiber (Table 1). Although previous studies have documented the benefits of feeding fermented banana stems or agro-waste to pigs [27,28,29], our current study provides enhanced scientific validation. As shown in Table 2, the BAWS contained volatile fatty acids, mainly acetic acid and lactic acid reduced the pH value, facilitated better preservation, as well as provided a satisfactory Flieg’s score of 54, confirming its suitability as preserved feed. Overall growth performance (Table 3) and the carcass, meat, and sensory scorings (Table 4) basically demonstrated no significant differences among groups, which supports the hypothesis in the recycling utilization of banana agro-waste in the pig industry. In addition, the production benefits post digestive adaptation, as with the initially lower daily weight gains, feed intake, and higher feed efficiency in BAWS groups (particularly 10%) were improved over time. Moreover, it was noteworthy that the reduced back fat and increased lumbar meat area of BAWS groups afforded the critical control of lean/fat ratio during the finishing stage of the pig industry.

### 4.2. Gut Microbiome of KHAPS Pigs and Populations Shift with Dietary BAWS

In the current study, microbiome analysis of fore gut and hind gut samples Via 16S rRNA sequencing confirmed sufficient sequencing depth and data quality. The KHAPS black pig fecal 16S rRNA phylogenetic analysis has been previously reported, and a novel species of *Micrococcus* was identified and cultured [30]. In this study, prevalence at the family level of both fore and hind guts revealed predominance of phyla of Bacillota (former *Firmicutes*), class of Clostridia, which includes two major families, *Clostridiaceae* and *Peptostreptococcus* (Figure 2). In agreement with others [31,32,33], over 60% of pig gut microbiota belonged to the class of Clostridia and over 80% to the phylum of Bacillota. It has been reported that Tibetan pigs fed on a nutrient-satisfying, formulated corn-based diet have their dominant phyla in colonic digesta were Bacillota, Bacterioideta, Spirochaetota, and Actinobacteriota [10]. Partially in agreement with their results, the top three phyla were the same in the present KHAPS pig study, while the Euryarchaeota was the fourth abundant phylum, followed by the fifth, Actinobacteriota. In the KHAPS breed, the archaeal phylum *Euryarchaeota* was found in relatively high abundance, and further analysis attributed this enrichment to the presence of the methane-producing *Methanobacteria* class. Yang et al., in their mini-review of swine gut archaea, highlighted the significance of such unique microbial populations for host energy metabolism, identifying them as economically important traits [34]. Moreover, the high abundance of *Methanobacteria*/*Euryarchaeota* in KHAPS pigs suggests substantial potential for further investigation, especially since this phylum was not detected in Rahman et al.’s NGS microbiome analysis of Canadian wild (*Sus scrofa*) and domestic (*Sus domesticus*) pigs using fecal swabs or colon digesta [35]. Nevertheless, the fecal swab microbiome analysis of the native Chinese Lan-tang pig, conducted in parallel with Duroc pigs, identified two archaea taxa, *Methanobarevibacter_A* and *Methanobarevibacter_A smithii*, within the phylum Euryarchaeota [33]. In this study, Euryarchaeota was also the least abundant phylum among the top 15 detected.

The relatively high and consistent abundance of *Clostridiaceae* in both the fore gut and hindgut across all experimental groups was noteworthy. Adult pigs at the finishing stage fed a nutrient-balanced complete ration (based on NRC guidelines) exhibited competent potential for fiber utilization, which was supported by their rich abundance of *Clostridiaceae,* particularly when compared with most other monogastric species. In the review by Yang et al. [36] has pointed out that the unique metabolic physiology of *Clostridium* functions like a cellular factor, enabling the sustainable breakdown of complex feedstocks’ fiber. Moreover, Gram-positive *Lactobacillus* has been reported as a dominant genus in the pig duodenum, where acidity is high [32]. With this in mind, the present study identified *Terrisorobacter* and *Clostridium_sensu_stricto_1* as the two dominant genera within the class Clostridia in the fore gut. Their increased abundance corresponded with a decrease in the *Lactobacillus* population in BAWS-fed pigs compared with controls (Figure 2). Previous studies have reported a significant reduction in the abundance of *Clostridium sensu stricto 1* under low-protein diets [32]. Moreover, Tibetan pigs fed an alfalfa-based diet that was associated with improved fiber utilization and short-chain fatty acids (SCFA) production exhibited altered colonic microbiomes. These microbiomes were dominated by genera of *Clostridum_sensu_stricto_1* and *Terrisporobacter* [10]. Based on the limited information, the present study is the first to report a diet-induced shift in fore gut microbiota, from health-associated *Lactobacillus* to fiber-degrading *Clostridum_sensu_stricto_1* and *Terrisporobacter* (Figure 2), following the inclusion of complex carbohydrate in a nutritionally balanced diet. Conversely, in the hind gut, the *Clostridium_sensu_stricto_1*, *Terrisorobacter,* and *Lactobacillus* were comparatively steady between BAWS and control groups, while a significantly increased relative abundance of the *Streptococcus* in BAWS groups was noticed (Figure 3). At the phylum level, both *Streptococcaceae* and *Prevotellaceae* exhibited increased abundances in the hindgut of the BAWS group, likely reflecting microbial adaptation to BAWS-derived nutrients. The genus *Streptococcus* was significantly increased in BAWS groups. *Streptococcus* is capable of metabolizing diverse carbon sources and amino acids, potentially aiding host energy balance. *Prevotella* is a dominant genus in plant-based diets and is capable of degrading hemicelluloses and complex polysaccharides. It is also one of the most abundant genera in the large intestine, where it contributes to amino acid metabolism, SCFA production, and vitamin K synthesis [32]. Furthermore, in weaned pigs receiving tryptophan supplementation, *Prevotella* abundance increased, which has been linked to immune enhancement via tryptophan metabolites [37]. The increase in anaerobic family Prevotellaceae with BAWS feeding aligns with prior findings, since banana peel and pseudostem are known to be rich in tryptophan. Whether dietary BAWS pose any health benefit in supporting gut microbiome homeostasis, tryptophan metabolism, or disease prevention may be of interest for future study.

### 4.3. Clusters of Orthologous Genes (COG) Database for Microbial Genome Annotation on Predicted Functional Proteins

In the hindgut, proteins represented by COG0534, COG2207, COG1476, COG1131, and COG1132 were significantly more abundant. These COGs are primarily associated with defense mechanisms, transcriptional regulation, and multidrug resistance. These proteins are critical for cellular adaptation and survival under stress. As to the fore gut groups, the COG2814 and COG1113 had higher relative abundance than the hind gut groups; these proteins are important in nutrient transport and metabolism to ensure metabolic balance and nutrient availability [38]. In the current study, the animals fed either the control or BAWS substituted diets were nutrient nutrient-balanced formula in consideration of protein, energy, and fiber levels. Inclusion of BAWS by substitution of corn meal had altered the protein and fiber compositions. Interestingly, 5% BAWS group displayed the lowest fore gut abundances of not only COG2814, but also COG0561, COG1131, and COG1132. When host energy status is relatively sufficient, the gut microbiome may adjust its activity to match the host’s metabolic state by reducing the abundances of these proteins, as the microbiome may rely less on nutrient scavenging or stress responses. In addition, if the host was under an energy-replete state, it might favor the proliferation of microbes adapted to nutrient-rich conditions, potentially leading to a reduction in proteins associated with nutrient acquisition or stress adaptation [39,40].

According to the National Center for Biotechnology Information (NCBI) COG database, COG0531 encodes a conserved protein domain associated with NADPH-dependent redox reaction. It may participate in the detoxification of azo, quinone, and other oxidized compounds Via reduction. COG0531 has also been implicated in amino acid transport and plays an important role in both absorption and metabolism. Although COG0531 abundance showed little variation across hindgut treatment groups, clear differences were observed in the fore gut microbiome between control and BAWS-fed pigs. The higher fore gut abundance of COG0531 in control pigs compared with BAWS-fed animals may reflect an increased need for oxidative stress management and amino acid utilization in the control diet. Hence, it may be assumed that dietary BAWS may confer enhanced oxidative stress protection and improve energy utilization from carbohydrates. Although its precise function remains unclear, COG2814 has been linked to metabolic pathways involved in cellular responses to environmental changes. It is also predicted as an arabinose efflux permease closely related to cellular metabolism and energy balance. Together with the higher abundances of COG0765, COG1113, COG1132, and COG0561, which are predicted to participate in environmental, metabolic, and ecological response pathways, ranked among the top 10 differential proteins in the gut microbiome. Their abundances in the fore gut microbiome were lower in BAWS groups compared with those in the corn-based control diet. Since host nutritional status and oxidative physiology could directly impact the gut microbiome by tightly regulating digestive secretions and immune innervation Via aligning lymphatic tissues, the fore gut microbiome may therefore be more sensitive than that of the hind gut. Reduction in these proteins in the gut microbiome might imply that the microbial environment is nutrient-rich, leading to reduced demand for amino acid transport and metabolism-related stress functions. Moreover, in the fore gut microbiome of BAWS-fed pigs, particularly those receiving 5% BAWS, there appears to be a notable reduction in *Lactobacillus* alongside an increase in *Clostridium* species (Figure 2). In line with this shift, proteins associated with nutrient acquisition and carbohydrate metabolism, such as COG2814 (an arabinose efflux permease), COG0561 (an L-asparagine transporter), and COG0765 (an ABC-type amino acid transporter), also tended to exhibit higher relative abundances in the fore gut of the 5% BAWS group (Figure 4). These observations may indicate that the microbial community adapted to favor taxa capable of breaking down fibrous substrates found in BAWS, equipped with molecular systems optimized for the uptake and metabolism of complex carbohydrates and amino acids. In contrast, the hind gut did not exhibit such genus-level shifts, and the abundances of these nutrient-related COGs remained relatively uniform across treatment groups. This indicates that dietary impact on functional potential was more pronounced in the fore gut, likely driven by early-stage digestion of BAWS components. Moreover, COG2207, encoding an AraC-type transcriptional regulator, was more abundant in BAWS-fed groups in the fore gut than in controls. Together, these results reveal a coherent correspondence: BAWS inclusion modulated both the taxonomic composition and the genomic functional profile of the fore gut microbiome, enhancing carbohydrate-processing and detoxification capabilities while stabilizing hind gut functions.

### 4.4. Study’s Limitations

Although the present study demonstrated that dietary inclusion of BAWS had potential benefits in finishing pig feeding, certain limitations should be acknowledged. Firstly, the consistency of BAWS composition between batches remains a key factor for industrial applications. Even with controlled fermentation conditions, the 30-day fermentation period requires considerable storage capacity for scaled-up production. Improving the efficiency of biotransformation and stabilizing the nutritional and microbial profiles of the final product would enhance its practicality. Secondly, while the study identified shifts in gut microbiome composition associated with nutrient metabolism, the potential synbiotic or postbiotic effects of BAWS, particularly its role in modulating host microbial colonization and health, remain to be clarified. Thirdly, functional prediction based on 16S rRNA gene sequencing relies on database-dependent inference tools such as PICRUSt2 and Tax4Fun2, which are limited in their ability to resolve metabolic functions at the species or strain level. Future studies incorporating metagenomic or metabolomic analyses may offer deeper insight into microbial functionality and host–microbiota interactions.

## 5. Conclusions

Fermentation effectively doubled crude protein in BAWS and produced short-chain fatty acids, achieving a satisfactory Flieg’s score. Dietary inclusion of up to 10% BAWS as a corn replacer in finishing pig diets proved nutritionally viable, with no adverse effects on performance or carcass traits. Microbiome and COG analyses revealed fore gut microbial restructuring and enhanced nutrient/stress response functions. BAWS may improve sustainability while maintaining production efficiency. Further studies are needed to evaluate long-term impacts on immunity, metabolism, and microbiota–host interactions.

## Figures and Tables

**Table 1:** Composition of experiment diets (as-fed basis).

Ingredient (%)	Control	5% BAWS	10% BAWS
**Corn meal**	68.09	63.09	58.09
**Soybean meal, 44%**	21.94	21.89	21.91
**Banana agro-waste Silage BAWS**	0.00	5.00	10.00
**Wheat bran**	3.00	2.31	1.53
**Dicalcium phosphate**	0.31	0.31	0.33
**Ground limestone**	0.81	0.82	0.83
**Molasses**	3.00	3.00	2.70
**Salt**	0.30	0.30	0.30
**Soybean oil**	2.27	3.00	4.04
**Vitamin premix ^1^**	0.10	0.10	0.10
**Mineral premix ^2^**	0.15	0.15	0.15
**L-Lysine-HCl (78%)**	0.03	0.03	0.03
**Feed Analysis composition (dry matter basis)**			
**ME, Kcal/kg**	3250.58	3250.19	3250.43
**Crude protein, %**	15.56	15.56	15.57
**Crude fiber, %**	1.95	1.88	1.80
**Ca, %**	0.68	0.69	0.68
**P, %**	0.52	0.53	0.51
**Lysine, %**	0.98	0.98	0.99
**Methionine, %**	0.31	0.31	0.31
**Threonine, %**	0.60	0.60	0.60
**Sulfur-containing AA %**	0.56	0.57	0.56

^1^ Vitamin supplied the following per kilogram of premix: Vitamin A, 5000 IU; vitamin D3, 1500 IU; vitamin E, 40 mg; vitamin K, 3 mg; vitamin B1, 2.6 mg; vitamin B12, 0.04 mg; niacin, 35 mg; pantothenic acid, 23 mg. ^2^ Mineral supplied the following per kilogram of premix: Fe (FeSO_4__7H_2_O, 20.09% of Fe), 217 mg; Cu (CuSO_4__5H_2_O, 25.45% of Cu), 125 mg; Mn (MnSO_4__H_2_O, 32.49% of Mn), 40 mg; Zn (ZnSO_4_, 80.35% of Zn), 110 mg; Se (NaSeO_3_, 45.56% of Se), 0.36 mg; Co (CoSO_4__H_2_O, 32% of Co), 0.7 mg.

**Table 2:** The analysis of nutritional values of the major ingredients in the banana agro-waste silage (BAWS), and the silage quality and Flieg’s score.

Item/Analyse(%)	DM	CP	NDF	ADF	ADL	WSC	P	K	Ca	Mg	TOC	NH_4_-N (ppm)
Disqualified banana	15.81	6.68	9.09	5.31	1.95	3.83	0.11	2.27	0.09	0.24	55.09	139.7
Pseudostem	23.70	6.10	37.43	25.00	4.65	17.23	0.11	2.20	0.83	0.93	51.85	158.1
BAWS (whole mixture)	23.69	12.43	28.08	15.24	2.33	1.40	0.53	1.40	0.16	0.38	54.79	1911.6
Quality	pH		Acetic acid		Butyric acid		Lactic acid		Flieg’s score			
BAWS	4.33 ± 0.27		4.41 ± 0.26		ND		2.46 ± 0.69		54.0 ± 3.0			

Abbreviations: DM—dry matter; CP—crude protein; NDF—Neutral detergent fiber; ADF—acid detergent fiber; ADL—acid detergent lignin; WSC—water soluble carbohydrates; P—phosphorus; K—potassium; Ca—calcium; Mg—magnesium; TOC—total organic carbon; NH_4_-N—amonium nitrogen; ND not detecable.

**Table 3:** Effect of different levels of dietary banana agro-waste silage on the growth performance of experimental pigs at the finishing stage.

Finishing Period (Day)	Control Group	5% BAWS	10% BAWS	*p*-Value
Body weight (kg)				
0	78	78	78	0.999
21	90	90	89	0.9586
42	102	101	99	0.8188
70	117	116	113	0.6961
Average daily weight gain (ADG) (kg)				
0–21	0.57	0.56	0.52	0.599
21–42	0.54 ^a^	0.51 ^ab^	0.47 ^b^	0.0059
42–70	0.73 ^a^	0.72 ^a^	0.69 ^b^	0.0008
0–70	0.62	0.60	0.56	0.0613
Average daily feed intake (ADFI) (kg)				
0–21	2.40 ^a^	2.37 ^a^	2.31 ^b^	0.0002
21–42	2.84 ^b^	2.89 ^a^	2.90 ^a^	0.0017
42–70	2.91	2.90	2.92	0.4875
0–70	2.71	2.72	2.71	0.7852
Feed conversion ratio (FCR) (ADFI/ADG)				
0–21	3.97	5.01	4.48	0.662
21–42	5.29 ^b^	5.64 ^b^	6.19 ^a^	0.0007
42–70	3.99 ^b^	4.04 ^b^	4.29 ^a^	0.0006
0–70	4.43	4.59	4.88	0.1286

Means in the same row were the average of 12 pigs in each group, and the significances at *p*-value < 0.05 were presented with different superscripts.

**Table 4:** Effects of dietary banana agro-waste silage on carcass, meat quality, and sensory score.

Items	Control Group	5% BAWS	10% BAWS	*p*-Value
Carcass				
Live weight, kg	115	114	112	0.7507
Slaughter weight, kg	99	98	95	0.7406
Dressing %	86	86	85	0.9222
Carcass length, cm	101.1	100.0	100.8	0.9027
Back fat thickness, cm	3.29	2.84	2.63	0.1950
First rib fat	3.99	3.61	3.82	0.6475
Last rib fat	3.29	2.70	2.30	0.1139
Lumbar fat	2.59	2.21	1.77	0.0638
Meat quality				
Color score ^1^	3.7	3.5	3.7	0.5156
Marbling score ^2^	2.7	2.6	2.7	0.9142
Firmness score ^3^	3.1	3.4	3.1	0.4930
Lumbar meat area ^4^	4.98	6.06	6.02	0.4832
Senory score ^5^				
Flavor	3.23	3.20	3.22	0.9404
Juiciness	3.05	3.12	3.10	0.5089
Tenderness	3.44	3.37	3.15	0.2770

Data were the average of 12 pigs of each group, and the significance was determined at a *p*-value less than 0.05. ^1^ Color score: 1 = pale gray to 5 = dark purplish red; ^2^ Marbling score: 1 = devoid to practically devoid to 5 = moderately abundant or greater; ^3^ Firmness score: 1 = very soft and very watery to 5 = very firm and dry; ^4^ Lumbar meat area measured at Longissimus muscle area at the 10th rib as square inch. National Pork Producers Council, 1991. ^5^ Sensory score was 1 to 10 scale.

## Data Availability

The data supporting the findings of this study are available from the corresponding author upon reasonable request.
